# Attenuation of polyglutamine-induced toxicity by enhancement of mitochondrial OXPHOS in yeast and fly models of aging

**DOI:** 10.15698/mic2016.08.518

**Published:** 2016-07-26

**Authors:** Andrea L. Ruetenik, Alejandro Ocampo, Kai Ruan, Yi Zhu, Chong Li, R. Grace Zhai, Antoni Barrientos

**Affiliations:** 1 Neuroscience Graduate Program, University of Miami Miller School of Medicine, Miami, FL 33136, USA.; 2 Department of Neurology, University of Miami Miller School of Medicine, Miami, FL 33136, USA.; 3 Department of Biochemistry and Molecular Biology, University of Miami Miller School of Medicine, Miami, FL 33136, USA.; 4 Department of Molecular and Cellular Pharmacology, University of Miami Miller School of Medicine, Miami, FL 33136, USA.; 5 Molecular and Cellular Pharmacology Graduate Program, University of Miami Miller School of Medicine, Miami, FL 33136, USA.; 6 Human Genetics and Genomics Graduate Program, University of Miami Miller School of Medicine, Miami, FL 33136, USA.

**Keywords:** Saccharomyces cerevisiae, mitochondrial respiration, mitochondrial OXPHOS, mitochondrial biogenesis, polyglutamine toxicity, yeast chronological life span, Drosophila model, caloric restriction

## Abstract

Defects in mitochondrial biogenesis and function are common in many neurodegenerative disorders, including Huntington’s disease (HD). We have previously shown that in yeast models of HD, enhancement of mitochondrial biogenesis through overexpression of Hap4, the catalytic subunit of the transcriptional complex that regulates mitochondrial gene expression, alleviates the growth arrest induced by expanded polyglutamine (polyQ) tract peptides in rapidly dividing cells. However, the mechanism through which *HAP4* overexpression exerts this protection remains unclear. Furthermore, it remains unexplored whether *HAP4 *overexpression and increased respiratory function during growth can also protect against polyQ-induced toxicity during yeast chronological lifespan. Here, we show that in yeast, mitochondrial respiration and oxidative phosphorylation (OXPHOS) are essential for protection against the polyQ-induced growth defect by *HAP4* overexpression. In addition, we show that not only increased *HAP4* levels, but also alternative interventions, including calorie restriction, that result in enhanced mitochondrial biogenesis confer protection against polyQ toxicity during stationary phase. The data obtained in yeast models guided experiments in a fly model of HD, where we show that enhancement of mitochondrial biogenesis can also protect against neurodegeneration and behavioral deficits. Our results suggest that therapeutic interventions aiming at the enhancement of mitochondrial respiration and OXPHOS could reduce polyQ toxicity and delay disease onset.

## INTRODUCTION

Metabolic and mitochondrial abnormalities are a prominent feature of aging and neurodegeneration, which is not surprising, given the high-energy demands of neuronal function. Mitochondria perform multiple roles essential for cellular metabolism and physiology. Their main function is the conversion of energy stored in nutrients into chemical energy, aerobically, through oxidative phosphorylation (OXPHOS), which couples oxygen reduction by the mitochondrial respiratory chain (MRC) to ATP synthesis.

Neurodegenerative diseases are frequently caused by the gain-of-toxic-function of disease-specific proteins that misfold and oligomerize, affecting the integrity and function of selective neuronal systems. Despite toxic protein heterogeneity, mounting evidence suggest that mitochondrial dysfunction and oxidative stress occur early in all major neurodegenerative diseases 1, including α-synucleopathies, taupathies, and polyglutamine (polyQ) disorders. PolyQ disorders, including HD and spinocerebellar ataxias, are caused by a CAG codon repeat expansion in disease-specific genes resulting in the expression of misfolding/aggregation-prone proteins with expanded polyQ stretches. Mitochondrial dysfunction, altered mitochondrial dynamics and impaired axonal trafficking have been associated with the pathogenesis of polyQ diseases in human patients and several research model organisms 2,3. In addition, mutant huntingtin (Htt) may damage neurons directly by inducing mitochondrial depolarization and altering calcium homeostasis in patients and in mouse models 2. Finally, mutant Htt has been shown to alter mitochondrial function indirectly by inhibiting expression of the transcriptional co-activator PGC-1α (peroxisome proliferator-activated receptor (PPAR)-gamma-coactivator 1α, which regulates several metabolic processes including mitochondrial biogenesis and respiration when interacting with the respiratory factors NRF1 and NRF2 3.

Due to the complexity of proteotoxic neuronal death, development of suitable models is critical to dissect the precise mechanism/s in the disease process and the aging/disease relationship. Whereas several mammalian cell culture models have been developed over the past 15 years, the unicellular yeast model *Saccharomyces cerevisiae* and its powerful genetics has also been successfully used to study the protein aggregation associated with polyQ disorders 4,5,6,7. Although yeast cells lack many of the structural and functional hallmarks of neuronal cells that are critical for the progression of neurodegeneration, yeast models have provided a platform to elucidate the basic cellular mechanisms of toxicity triggered by human neurotoxic proteins and to identify targets for therapeutic intervention 7,8,9. In *S. cerevisiae*, expression of Htt exon-1 fragments comprising the polyQ stretches faithfully recapitulates Htt misfolding/aggregation in a polyQ length-dependent manner 10. Upon polyQ toxicity, several pathways, including ER stress, cytoskeletal disturbances, oxidative stress, and mitochondrial dysfunction contribute to growth arrest and cell death 4,6,11. Genetic or pharmacological interventions aiming to protect each of these pathways from disruption have been shown to confer protection against polyQ toxicity. Specifically, we have shown that in growing yeast cultures, polyQ-induced toxicity can be suppressed by enhancement of mitochondrial biogenesis, achieved by overexpression of Hap4, the catalytic subunit of the transcriptional complex Hap2,3,4,5 that globally activates transcription of nuclear genes involved in mitochondrial respiration 12. The specific mechanism involved in the suppression remains to be fully elucidated.

As a handicap for the currently available yeast models of proteopathies, all studies reported to date have been conducted on rapidly dividing mitotic cells, and have manifested acute toxicity based on the high galactose-inducible expression of toxic proteins. Only a single study has looked at yeast survival in the stationary phase of yeast expressing α-synuclein, also from a galactose-inducible promoter 13. Nevertheless, in this report, α-synuclein expression was induced at the moment of inoculation, severely restricting survival during the exponential phase and diauxic shift. While studies in dividing yeast are interesting and have provided useful information into the diverse cellular processes that mutant proteins can alter, these models fail to mirror the post-mitotic state of neurons in the adult brain. Furthermore, due to the fact that neurodegenerative disorders are age-associated disorders, we believe that disease models that facilitate the study of proteotoxicity in the context of aging will be more suitable for the better understanding of these kinds of diseases. These considerations support yeast chronological aging as a good model to study mitochondrial function alterations involved in aging and age-related disorders 8.

The yeast chronological life span (CLS) model of aging measures the capacity of stationary (G_0_) cultures to maintain viability over time (1 to several weeks) 14**.** The CLS model of aging is an established model for the regulation of aging in post-mitotic mammalian cells, such as neurons 8. For CLS studies, yeast cells are usually aged in media containing 2% glucose. Under these conditions, cells divide exponentially producing energy preferentially by fermentation while respiration is repressed in a glucose concentration-dependent manner. As glucose is being consumed, growth slows down and the diauxic shift occurs, which involves a shift from fermentation to respiration. In addition, the activation of stress resistance mechanisms and the accumulation of nutrient stores (glycogen and trehalose) allow the survival in the stationary phase where the metabolic rate is significantly reduced. Mitochondrial respiration during growth is known to be essential for a strain to achieve a standard wild-type CLS 15. However, yeast cells have a large reserve respiratory capacity to sustain CLS, as respiration only limits CLS when depleted below a threshold of ~40% of wild-type respiration 16. Strains that respire below this threshold during growth have extremely poor respiratory capacity in the stationary phase and rapidly consume their nutrient stores due to the inefficient production of energy through fermentation, resulting in a very short CLS 16. How polyQ expression interferes with yeast CLS remains unclear.

To study polyQ toxicity in the context of aging and its suppression by enhancement of mitochondrial biogenesis, we have created flexible beta-estradiol inducible yeast models that allow for the tight regulation of toxic protein expression at any moment of growth. Our data show that boosted mitochondrial respiration during growth is essential for Hap4-induced suppression of polyQ toxicity during CLS and that several genetic and nutritional interventions that enhance mitochondrial respiration during growth and extend life span effectively alleviate polyQ toxicity during CLS. These data has been subsequently validated in a *Drosophila* model of HD, where overexpression of a PGC1-α homolog also protects against neurodegeneration and behavioral deficits. Our results further suggest that therapeutic interventions that enhance mitochondrial OXPHOS could reduce polyQ toxicity and delay disease onset in patients.

## RESULTS 

### Development of yeast models of polyQ cytotoxicity in post-mitotic cells. 

**Figure 1:**
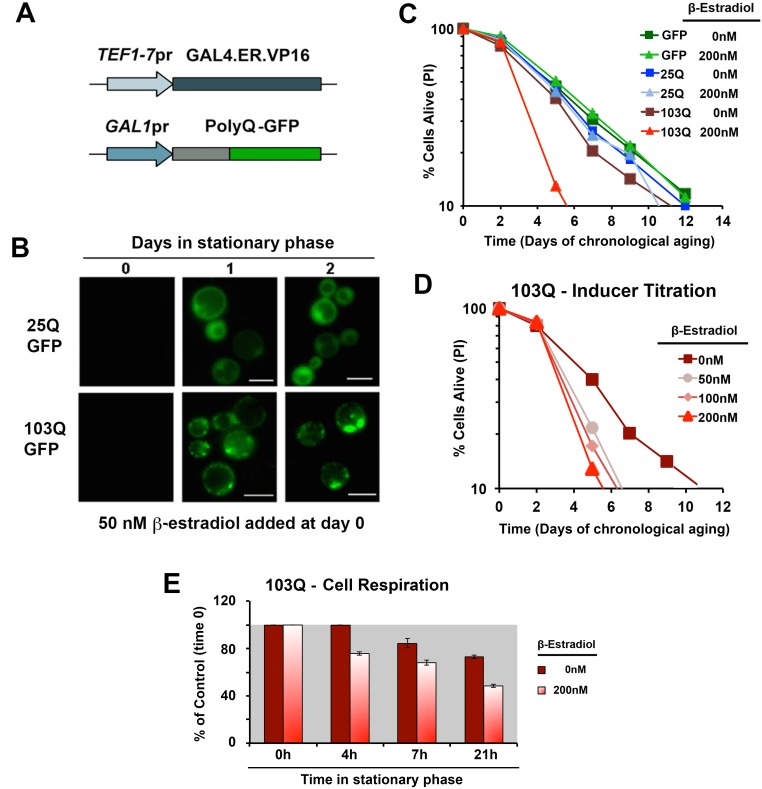
FIGURE 1: Generation of inducible yeast models of proteopathies. **(A)** Schematic of polyQ-GFP expression cassettes under the control of the β-estradiol inducible promoter. **(B)** Chronology of polyQ-GFP protein accumulation, followed by fluorescence microscopy, in cells induced with 50 nM β-estradiol. The bar is 5 µm. **(C) **and** (D)** Yeast CLS. Survival of wild-type cells expressing 25Q or 103Q from a β-estradiol-inducible promoter activated with the indicated amounts of inducer or supplemented with the solvent (ethanol) was estimated by propidium iodide (PI) staining and flow cytometry analysis of 10,000 cells. Data is an average of three samples in % of cells alive at day 0 (72 h after inoculation). In **(D)** a β-estradiol titration was performed. **(E) **Time-course of endogenous cell respiration during the first day in stationary phase of growth of non-induced and induced 103Q cultures.

In order to study proteotoxicity in post-mitotic yeast using the CLS assay, we generated metabolism-independent inducible models of polyQ toxicity. To this end, we constucted and tested several candidate expression systems 17, and ultimately chose an optimized β-estradiol-inducible system modified from 18 as the system that performed best in our conditions (**Figure 1A**). This expression system is based on the constitutive expression of a transactivator fusion protein GAL4.ER.VP16, which is formed by a *GAL4* DNA binding domain, a β-estradiol receptor domain and a VP16 (virus protein 16) transcriptional activator that can activate transcription of a gene placed under the control of a galactose-inducible promoter (*GAL1*pr). In the absence of the hormone, the fusion protein is repressed by the yeast chaperones from the Hsp90 family 18. Upon media supplementation with β-estradiol, the fusion protein is de-repressed, binds to *GAL1*pr through the *GAL4* DNA binding domain and the VP16 recruits the transcriptional machinery to start transcription of the gene of interest.

Using this system, we created models expressing 25Q, 46Q, 72Q or 103Q N-terminal Huntingtin (Htt) exon-1 fragment proteins, and have confirmed that polyQ expression is well-induced with a low β-estradiol concentration (50 nM) upon transition to the stationary phase (see 25Q and 103Q in **Figure 1B**) without detectable leakage of the system before induction (**Figure 1B** and 17). For the chronological life span (CLS) experiments described herein, wild-type cells expressing 25Q- and cells expressing highly-pathogenic 103Q-N-terminal Htt, under the control of the described β-estradiol inducible promoter, were pre-grown in minimum media containing 2% glucose until they reached stationary phase (72 hours). At this moment, protein expression was induced by addition of β-estradiol. After induction, polyQ protein accumulates in a diffused distribution in the cytoplasm of 25Q-expressing cells, while 103Q fragments form insoluble protein aggregates over time (**Figure 1B**). When expression was induced with 200 nM β-estradiol, expression of GFP or 25Q did not produce any measurable effect in CLS, as determined by flow cytometry after PI staining, whereas 103Q produced a toxic effect that curtails maximum yeast CLS by 50%, (**Figure 1C**). These results were in agreement with the analysis of cellular viability using a clonogenic assay based on counting of CFU (colony formation units, **Figure S1**). A titration of the inducer indicated that polyQ expression is well induced and only slightly increases further in a dose-dependent manner with concentrations from 50 nM to 200 nM, although 103Q toxic effect on CLS was similar in this concentration range (**Figure 1D**). As a control, no toxic effects of the hormone were observed (not shown).

### PolyQ cytotoxicity in chronologically aging yeast is suppressed by genetically-driven enhancement of mitochondrial biogenesis 

We have previously reported that cytotoxicity of a mutant huntingtin fragment in yeast involves early alterations in mitochondrial OXPHOS function, loss of membrane potential and ROS generation 4,5. Here, we have observed that 103Q expression induced at day 0 of chronological life span resulted in 25% lowered endogeneous cell respiration after 4 h in stationary phase, when all the cells are still alive (**Figure 1E**). Although respiration of non-induced cells also decreased over time, induced cells were decreased by 35% after 20 h in stationary phase (**Figure 1E **and** 2A**). As a toxicity suppressor mechanism, we have shown earlier that enhancement of mitochondrial biogenesis through overexpression (OE) of Hap4, the catalytic subunit of the HAP transcriptional complex that serves as the master regulator of nuclear genes encoded mitochondrial proteins in yeast 12, confers robust protection against the 103Q-induced growth deficit during the exponential phase 4. Therefore, we hypothesized that *HAP4* may also confer protection against 103Q-induced toxicity in the stationary phase.

**Figure 2:**
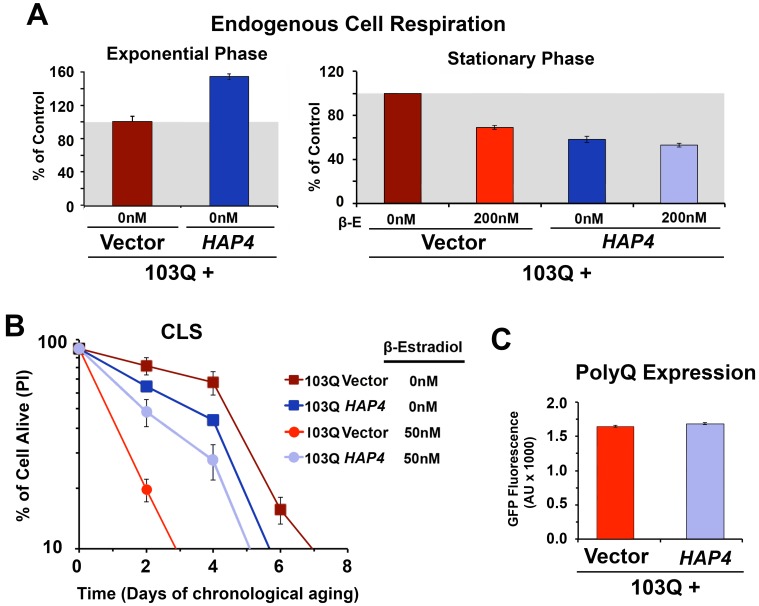
FIGURE 2: PolyQ-induced toxicity in CLS models of yeast aging is suppressed by *HAP4* overexpression-mediated increase in mitochondrial biogenesis. **(A)** Endogenous cell respiration during the exponential and stationary phases of growth of non-induced and induced 103Q cultures overexpressing or not *HAP4*. Bars represent mean ± S.D., n = 3. **(B)** Effect of increased mitochondrial biogenesis by *HAP4* overexpression on CLS of yeast expressing 103Q from day 0 in the stationary phase. Error bars represent S.E.M. for 3 independent experiments. **(C)** PolyQ expression levels at day 1 after β-estradiol induction estimated by flow cytometry quantification of GFP fluorescence.

To test this hypothesis, 103Q cells and 103Q cells carrying a construct that overexpresses *HAP4* under the control of its own promoter were grown in media containing 2% glucose and 103Q expression was induced upon transition to the stationary phase. As previously reported 16, non-induced wild-type-like cells constitutively overexpressing *HAP4 *respired at a rate of 150% of wild-type during growth and 50% of wild-type during the stationary phase (**Figure 2A**). For clarification, wild-type strains have a reduced metabolic rate when they reach the stationary phase, and their respiratory rate at day 0 of CLS (72 hr after inoculation) - here considered as 100% - is actually 20% of their exponential-phase respiratory rate 16.

*HAP4* OE was previosuly reported to slightly extend CLS when the cells were transferred to water upon reaching the stationary phase 19. However, under our experimental conditions, enhanced mitochondrial biogenesis and respiration during growth was not sufficient to extend maximum CLS in non-induced cells 16 (**Figure 2B**). On the contrary, enhanced mitochondrial biogenesis was able to prevent the decline in respiratory capacity of 103Q-expressing cells after 20 hours in exponential phase (**Figure 2A**) and, most importantly, provide a CLS extension to yeast expressing 103Q, from 3 to 5 days (**Figure 2B**). This suppression of 103Q toxicity was not due to decreased 103Q protein, as polyQ-GFP fluorescence was indistinguishable between cells with or without *HAP4 *expression (**Figure 2C**). We envision that by increasing mitochondrial biogenesis during growth, the cell becomes equipped with a larger buffering system against 103Q-induced mitochondrial damage later during CLS 4. Furthermore, preserving aerobic energy conversion is expected to have a positive impact in ATP-dependent 103Q clearance pathways and refolding chaperone systems.

### PolyQ cytotoxicity in chronologically aging yeast is attenuated by nutritional respiratory preconditioning 

Yeast CLS has been reported to be extended by increasing the levels of cellular respiration during the initial exponential growth phase achieved by culture on a respiratory carbon source 4,16,19,20,21,22 or by pre-culturing the cells in media containing 0.5% rather than 2% glucose (caloric restriction or CR) 16,23,24,25. Although the mechanisms involved in the two models are very different, as explained below, we wanted to test whether suppression of 103Q toxicity during CLS could also be confered by these interventions known to enhance mitochondrial respiration and biogenesis.

**Figure 3:**
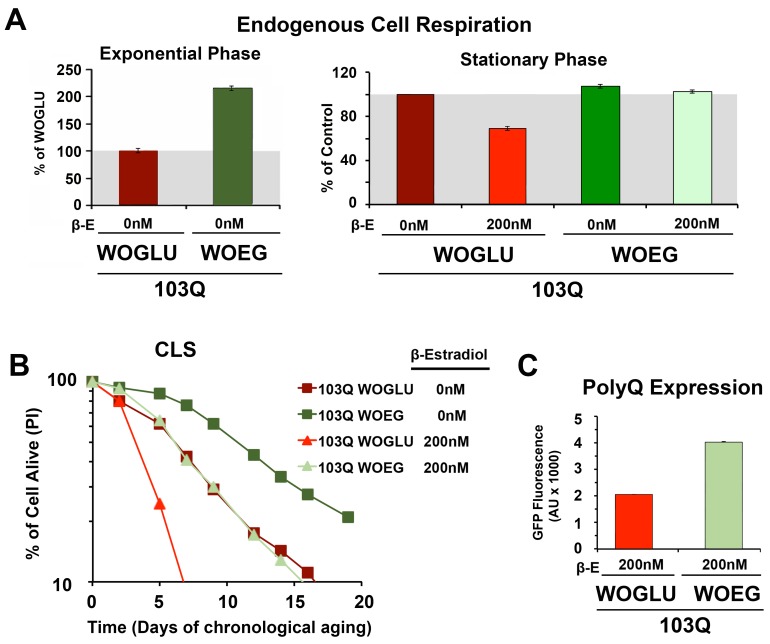
FIGURE 3: PolyQ-induced toxicity in CLS models of yeast aging is minimized by pre-conditioning the cells in respiratory media. **(A)**Endogenous cell respiration during the exponential and stationary phases of growth of non-induced and induced 103Q cultures grown in minimum synthetic media containing either the fermentable glucose (WOGLU) or the non-fermentable ethanol-glycerol (WOEG) as the carbon sources. Error bars are S.D., n = 3. *P <0.05, **P <0.005. **(B)**Effect of growth in WOEG respiratory media on 103Q yeast CLS compared to WOGLU media. S.D < 1 for all samples, n = 3. **(C) **PolyQ expression levels at day 1 of CLS after β-estradiol induction estimated by flow cytometry quantification of GFP fluorescence.

First, we started by modifying the synthetic medium from containing glucose (WOGLU), a fermentable carbon source, to containing the non-fermentable carbon sources ethanol and glycerol (WOEG). In the presence of glucose, mitochondrial biogenesis is repressed in a concentration-dependedt manner through inhibition of the HAP complex 26. On the contrary, growth in WOEG media results in HAP-mediated increase in mitochondrial biogenesis, including components of the mitochondrial respiratory chain and oxidative phosphorylation system 27,28, similar to what is achieved by overexpression of *HAP4*. This was reflected by a 2-fold higher endogenous cell respiration during growth in WOEG than in WOGLU (**Figure 3A**). Similarily to the results obtained with *HAP4 *OE, growth in WOEG media suppressed 103Q toxicity by preventing the decline in respiratory capacity of 103Q-expressing cells after 20 h in exponential phase (**Figure 3A**) leading to a 2.3-fold increase in maximum survival of 103Q-expressing cells (**Figure 3B**). This protective effect was not due to a reduction in the levels of polyQ protein that were actually higher in WOEG media compared to WOGLU, as estimated by GFP fluroescence (**Figure 3C**). The CLS extension conferred by growth in respiratory carbon sources has been suggested to be a reflection of efficient survival in stationary phase requiring respiratory metabolism 29 and a reduction in medium acidification 21. Furthermore, respiration-adapted cultures, do not just display maximal longevity, but also maintain full replicative lifespan during CLS 19. Regarding 103Q-expressing cells, we envision, as for *HAP4* OE cultures, that enhancement of mitochondrial biogenesis and respiration, could minimize the effect of 103Q-induced mitochondrial damage and might enhance the activity of 103Q refolding chaperone systems.

**Figure 4:**
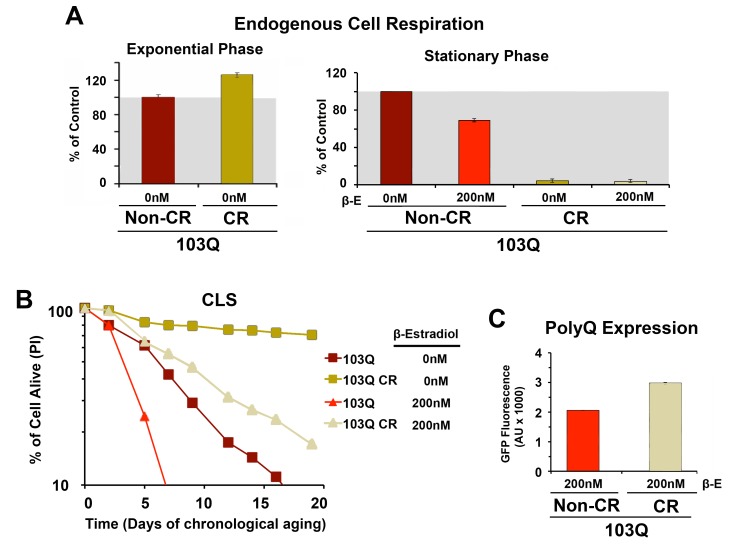
FIGURE 4: PolyQ-induced toxicity in CLS models of yeast aging is markedly suppressed by pre-conditioning the cells in 0.5% glucose-containing media (caloric restriction or CR). **(A)** Endogenous cell respiration during the exponential and stationary phases of growth of non-induced and induced 103Q cultures grown in minimum synthetic (WOGLU) media containing either 2% (control) or 0.5% (CR) glucose as the carbon source. Bars represent mean ± S.D., n = 3. **(B)** Effect of CR in 103Q yeast CLS. S.D < 1 for all samples, n = 3. **(C) **PolyQ expression levels at day 1 after β-estradiol induction estimated by flow cytometry quantification of GFP fluorescence.

Subsequently, we decided to test whether caloric restriction (CR) could also suppress 103Q toxicity. In yeast, CR, modeled by growing cells in media with 0.5% glucose, has been shown to result in a Hap4-dependent enhancement of mitochondrial biogenesis, as well as an increase in respiration (**Figure 4A** and 16) and ROS-adaptive signaling during growth 16. CR extends CLS in part through the downregulation of signaling pathways involving nutrient-responsive kinases, such as Ras/cAMP/PKA, TOR, and Sch9, which promote the expression of stress-response genes and the accumulation of storage carbohydrates 16,23,25. We recently reported that CR-induced extension of CLS required the cells to have a minimum threshold respiratory capacity (40% of wild-type) during exponential growth in non-restricted WOGLU media 16. In wild-type cells 16 or in our non-induced 103Q cells (**Figure 4A**), CR led to a 30% higher respiratory rate during exponential growth but, importantly, completely abolished the need for respiration in the stationary phase. CR involves a metabolic remodeling that includes the shift from fermentation to respiration, which is essential for CLS extension 24. However, the metabolic remodeling that occurs during the diauxic shift allows CR cells to dramatically reduce their metabolic rate in the stationary phase and hence consume their stored nutrients at a rate slower than that of cells grown in 2% glucose 16,30. Furthermore, in stationary phase, CR cells have lowered oxidative stress 16,20, which may contribute to CLS extension.

As expected, CR-treated non-induced cells had a markedly longer lifespan than those grown without the intervention (**Figure 4B**), which is in agreement with previous reports showing that, in wild-type cells, CR led to a more than 2-fold extension of their maximum CLS to 27 days 16,23. Induction of 103Q-expression at day 0 of CLS in CR-treated cells was still toxic and reduced maximum CLS to slightly over 20 days. However, attention should be focused on the fact that 103Q-expressing cells grown in CR media increased cell survival from 7 days to more than 20 days, an extenstion over that of even non-induced cells growing in non-CR medium (**Figure4B**).

Because mitochondrial respiration is not required for CLS in CR-treated cells, our data indicate that 103Q toxicity during CLS involves multiorganelle pathways as it does in growing cells. As for the anti-toxicity interventions discussed in previous paragraphs, the CR-treatment effect was not due to decreases in polyQ protein, since, like for cells grown in WOEG, polyQ expression was estimated to be significantly higher in CR cells than those grown in standard glucose-containing media (**Figure 4C**).

### Mitochondrial respiration is essential for suppression of PolyQ toxicity via enhanced mitochondrial biogenesis

Although all the anti-103Q toxicity interventions presented in the previous sections involve increases in cellular respiration during growth, we wanted to further explore whether the mitochondrial contribution to this protection involves OXPHOS or if it may relate to some other pathway affected by enhancement of mitochondrial biogenesis. In order to test whether mitochondrial respiration is required for *HAP4*-mediated suppression of 103Q proteotoxicity, we turned to our mitotic models of galactose-induced polyQ expression during exponential growth. For these assays we used strains expressing either non-toxic 25Q or toxic 103Q motifs further modified to overexpress *HAP4 *or to carry an empty episomal plasmid (EP). Here, we abolished mitochondrial OXPHOS function by genetic and pharmacologic manipulations and determined the efficacy of *HAP4*-mediated suppression of 103Q-induced cytotoxicities.

**Figure 5:**
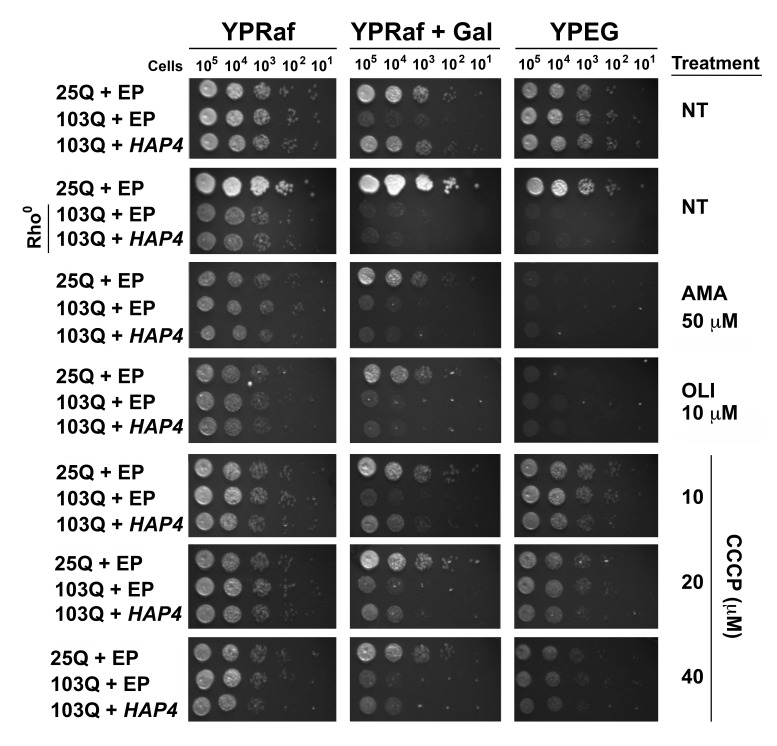
FIGURE 5:Mitochondrial respiration is essential for suppression of polyQ-induced toxicity by *HAP4* overexpression. Serial dilutions of yeast strains on non-inducing media (YPRaf), polyQ-inducing media (YPRaf+Gal), and respiratory media (YPEG) after 2 days of growth at 30°C. Rho^0^ indicates cells without mitochondrial DNA. EP, Empty plasmid; NT, no treatment (NT); AMA, antimycin A; CCCP, Carbonyl cyanide m-chlorophenyl hydrazone; OLI, oligomycin.

First, we used previously reported 103Q yeast, with or without the overexpression of *HAP4*, that had been completely depleted of mitochondrial DNA (mtDNA) and therefore are rho^0^ strains 31. The mtDNA encodes essential components of the OXPHOS system, and, therefore, rho^0^ yeast are unable to grow in complete media in the presence of respiratory substrates such as ethanol and glycerol (YPEG). Because rho^0^ cells do not grow efficiently in galactose-containing media, we used the non-repressible raffinose as the fermentable carbon source and induced protein expression when required by supplementing the plates with 0.25% galactose. As shown in **Figure 5**, *HAP4* OE suppressed 103Q-induced toxicity, as previously reported 4. As expected, the suppression was only effective in rho^+^ strains, containing mtDNA, but not in rho^0^ strains, suggesting that *HAP4*-mediated suppression requires functional mitochondrial respiration.

In addition to the genetic approach, we also pharmacologically inhibited mitochondrial respiratory complex III activity with antimycin A (AMA) that prevents growth in respiratory media (YPEG). AMA treatment abolished the suppression by *HAP4* OE (**Figure5**). Similar outcomes were obtained when mitochondrial OXPHOS was impaired by supplementation of the growth media with oligomycin (OLI), an inhibitor of the F_1_F_o_ ATPase. We next treated the cells with increasing concentrations of the ionophore carbonyl cyanide m-chlorophenyl hydrazine (CCCP). CCCP causes an uncoupling of the mitochondrial proton gradient from ATP production, causing a futile increase in mitochondrial respiration. Although still effective, a reduction in the protective effects of *HAP4* OE in 103Q-expressing cells was seen with increasing concentrations of CCCP (**Figure 5**). These results strongly indicate that coupled mitochondrial respiration is the essential component of the *HAP4 *mechanism of protection against proteotoxicity in yeast.

**Figure 6:**
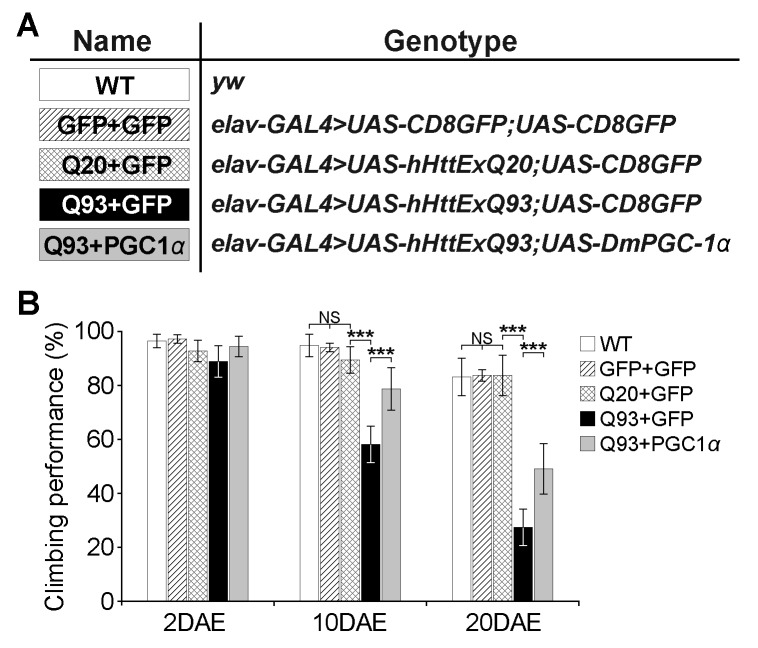
FIGURE 6: DmPGC-1α/spargel protects against hHttExQ93-induced climbing defects. **(A)** List of genotypes of the transgenic flies examined. The first exon of human huntingtin gene with 20 (UAS-hHttExQ20) or 93 (UAS-hHttExQ93) repeats of polyQ, and GFP (UAS-CD8GFP) or DmPGC-1α/spargel (UAS-DmPGC-1α) were expressed by the pan-neuronal driver *elav^C155-GAL4^*. **(B)** Climbing performance of wild-type, GFP over-expressing flies, and hHtt-expressing flies that co-expressed GFP or PCG-1α at the ages of 2, 10 and 20 DAE (days after eclosion). Ten groups (10 flies in each, total one hundred flies) of each genotype and age were tested. All data were presented as mean ± S.D. n = 10. Significance level was established by One-Way ANOVA post hoc Tukey’s test. *** P < 0.001.

### Attenuation of PolyQ-induced neurodegeneration in a fly model of Huntington’s disease via enhanced mitochondrial biogenesis

**Figure 7:**
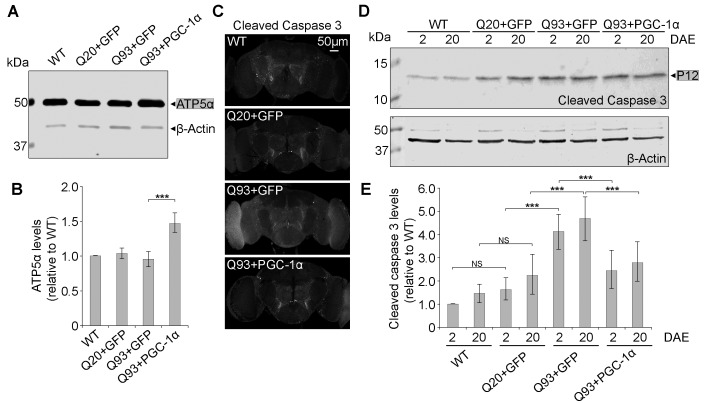
FIGURE 7:DmPGC-1α/spargel suppresses hHttExQ93-induced activation of cell apoptosis. **(A-B) **Western blot analysis** (A) **and quantification **(B)** of proteins extracted from 2 DAE wild-type, hHttExQ20- or hHttExQ93-overexpressing fly heads. ATP5α was used as a mitochondrial marker. β-Actin was used as an internal control. For quantification, fold change relative to the level of WT group is displayed. **(C) **Adult (2 DAE) female brains of wild-type, hHttExQ20- or hHttExQ93-overexpressing flies were probed for cleaved caspase-3. Scale bar is 50 µm. **(D-E)** Western blot analysis **(D)** and quantification **(E)** of proteins extracted from 2 DAE and 20 DAE wild-type, hHttExQ20- or hHttExQ93-overexpressing fly heads. For quantification, P12 was considered as cleaved caspase-3 (grey box): -fold change relative to each level in 2 DAE wild-type flies. β-Actin was used as an internal control. All data were presented as mean ± S.D., n = 3. Significance level was established by One-Way ANOVA post hoc Bonferroni test. *** P < 0.001.

To test whether our results in yeast could be replicated in higher organisms, we turned to a *Drosophila* model of HD. Similar to the function of the Hap2/3/4/5 complex in yeast, PGC-1α in higher organisms is a transcriptional activator critical in regulating mitochondrial biogenesis 25. In *Drosophila*, overexpression of the PGC-1α homolog (*DmPGC-1*/*spargel*) has been shown to be sufficient to increase mitochondrial OXPHOS activity, which leads to an extension of fly life span and improvement in tissue homeostasis in aged flies 32. Here, we used a pan-neuronal driver *elav^C155-GAL4^* to express the exon 1 of human *huntingtin* gene with pathological 93 polyQ repeats (hHttExQ93) or non-pathological 20 polyQ repeats (hHttExQ20) in the fly nervous system 33. First, we tested the locomotor activity of the flies by a negative geotaxis assay. As shown in **Figure 6**, while flies expressing hHttExQ20 showed similar climbing behavior as wild-type flies (*yw*), expression of hHttExQ93 caused an age-dependent decline of the climbing performance that was significant at 10 and 20 days after eclosion (DAE). This decline of climbing performance was suppressed by co-expression of DmPGC-1α. Consistent with a previous report 32, overexpression of DmPGC-1α enhanced mitochondrial biogenesis by 50% in fly brains, as monitored by the steady state levels of ATP5α, a subunit of mitochondrial F_1_F_0_-ATP synthase 34,35 (**Figure 7A-B**).

To examine the cellular effect of enhancing mitochondria biogenesis on mutant Htt-induced neurodegeneration, we determined the level of caspase-3 activation, a hallmark of apoptosis 36, at early (2 DAE) and late (20 DAE) ages. Neuronal expression of hHttExQ93 induced an increased level of cleaved caspase-3 throughout the entire fly brain at 2 DAE, which could be suppressed by co-expression of DmPGC-1α**(Figure 7C)**. Therefore, neuronal apoptosis occurred early following hHttExQ93 expression, before the onset of significant decline in climbing behavior. To further quantitatively monitor the activation of apoptosis, we measured the expression level of caspase 3 P12, the activated and fully processed product of pro-caspase 3 37. Consistently, expression of hHttExQ93 significantly increased P12 level in the fly brains, which could be attenuated by overexpression of DmPGC-1α at both 2 DAE and 20 DAE (**Figure 7D-E**).

Collectively, these results indicate that enhancing mitochondrial biogenesis by overexpressing DmPGC-1α protects against polyQ-induced neurotoxicity, and this neuroprotective mechanism is likely conserved between yeast and flies.

## DISCUSSION

The role of mitochondrial respiration and oxidative phosphorylation in proteotoxic neurodegeneration is a longstanding question, and the mechanism by which enhancement of mitochondrial biogenesis protects against proteotoxicity is still poorly understood. Polyglutamine toxicity is pleiotropic, affecting multiple pathways and organelles, including mitochondria. Among the mitochondrial pathways, our studies using yeast CLS and fly models of neurodegenerative proteopathies identify OXPHOS as the pathway that is most affected by polyglutamine expression and oligomerization. Notably, we reveal that genetic and nutritional interventions that enhance mitochondrial biognesis to precondition cells and tissues with high respiration serve to attenuate polyQ-induced proteotoxicity.

Most previous studies of proteotoxicity suppression in yeast models have used rapidly dividing mitotic cells, and have established acute toxicity with a high expression of toxic proteins using galactose-inducible promoters 4,5,6,7,38. Here, using a β-estadiol expression system capable of inducing toxic polyQ expression at the point of transition to stationary phase 17, we have documented an early mitochondrial respiratory defect. We have also shown that enhancement of mitochondrial biogenesis by overexpression of *HAP4* partially suppresses polyQ-induced toxicity in the stationary phase of the CLS assay, a model for neuronal aging, as previosuly reported for the polyQ-induced growth deficit in the exponential phase 4. Importantly, we report that, in the exponential growth model, anti-polyQ protection by *HAP4* overexpression is abolished with the inhibiton of mitochondrial respiration, even though the increase in mitochondrial biogenesis remains, thus demonstrating that protecting OXPHOS pathway is a key for polyQ toxicity attenuation. We have further exploited the yeast paradigm to demonstrate that growth in non-fermentable media and CR media, nutritional interventions known to increase mitochondrial biogenesis and mitochondrial respiration during growth, also confer protection against stationary-phase polyQ toxicity. In the case of CR, multiple additional pathways could be relevant. For example, despite the consensus that mutant polyQ- and alpha-synuclein-induced toxicities involve several distinct pathways, it has been recently reported that CR reduces alpha-synuclein toxicity in aged yeast cells by controlling the maintenance of autophagic activity at homeostatic levels 39. The possibility exist, however, that the control of autophagy in these growth conditions is a consequence of the CR-induced enhanced OXPHOS function. 

Over the several experimental settings presented here, we have observed that enhanced respiration during growth results in lowered endogenous cell respiration during stationary phase, the most extreme case being CR-treated cells, which survive without utilizing respiratory metabolism 16. We have previously shown that higher respiration during growth is associated with higher ROS generation, which can act as signaling molecules to induce an adaptive response in stationary phase involving metabolic remodeling and slow metabolism. This is particularly true for cells grown under CR conditions or in the presence of rapamycin to inhibit the TOR pathway 16,40, and it is also true for cells overexpressing *HAP4*^16^. 

We need to emphasize, however, that we performed our respiratory assays using cells in their culture media, and therefore we are measuring “real time” respiration. CR-treated cells maintain a high mitochondrial mass in stationary phase, but this capacity is not utilized for respiration during CLS. The relationship between high respiration during growth phases and chronological life span is also intriguing. An important example are the *puf3* mutant strains, which include an increased OXPHOS abundance and respiration during all phases of growth, but a wild-type CLS 41. In agreement with this, we have shown that increased respiration during growth (e.g., by *HAP4* OE) is not sufficient to extend CLS of wild-type cells if it is not accompanied by an enhancement of cell protection systems that promote survival in the stationary phase as for a *tor1* mutant- and CR-treated cells 16. However, in chronologically aged 103Q-expressing cells, in which mitochondrial function is altered, even *HAP4* overexpression is able to provide the enhanced mitochondrial mass buffer needed to overcome the polyQ-induced mitochondrial toxicity.

As an important validation of the observations made in the yeast models, we have confirmed our *HAP4* overexpression results in a fly model of HD, in which we have shown that overexpression of the *Drosophila *PGC-1α homolog (*DmPGC-1*/*spargel*) is also able to protect against the behavioral deficits and neurodegeneration induced by polyQ proteins. It is important to note the potential difference in hHttExQ93 expression levels between our study and previous studies using the same transgenic line 42. In our study, to ensure similar expression level of hHttExQ93 among all groups, we used *UAS-GFP* as a control transgenic element. Therefore, the level of hHttExQ93 expression in our study is likely lower than that in previous studies. As a result, the progression of degeneration is slower as hHttExQ93-expressing flies in our study all survived beyond 20 DAE, while a median lifespan of 10 DAE (maximum 17 DAE) was reported in the previous study 42. The slightly slower degeneration gave us the temporal resolution to assess the age-dependent decline in climbing behavior and the suppression by PGC-1α. Interestingly, we observed the activation of apoptosis before the onset of behavior defects, suggesting apoptosis as an early event and a likely cause of degeneration. Importantly, PGC-1α expression suppresses apoptosis from the early age, providing the molecular basis for the neuroprotection offered by PGC-1α. 

These results are in agreement with previous studies showing that transcriptional repression of PGC-1α by mutant Htt leads to mitochondrial dysfunction and neurodegeneration in HD mouse models and that exogenous expression of PGC-1α in this model protects against mutant Htt-induced neurotoxicity 3,43. Furthermore, PGC-1α has been proposed to be a modifier of onset age in HD patients, although functional studies are needed to confirm this and to identify the genetic variations in PGC-1α that enhance or alleviate HD pathogenesis 44,45,46.

In conclusion, our results in post-mitotic yeast, using the CLS assay, as well as our results in the *Drosophila *model of HD, shed new light on how mitochondrial respiration and OXPHOS-dependent pathways are affected by polyQ-induced toxicity, and support the need for further research into the possibile therapeutic potential of interventions aimed at increasing mitochondrial biogenesis in patients suffering from HD and other neurodegenerative proteopathies. 

## MATERIALS AND METHODS

### Yeast strains and methods

#### Yeast strain and media

The yeast strains used in this study were constructed from the previously reported 25Q and 103Q isogeneic strains, and the respective *HAP4 *overexpressing strains, with the *S. cerevisiae *W303-1A background 4,5, modified with the β-estradiol-inducible *TEF1-7* promoter described in 17. Compositions of the different growth media have been described 47,48,49. Prior to induction with galactose or the indicated amount of β-estradiol, all strains were grown in non-inducible media. For the chronological lifespan assay, strains were grown in the indicated media, using the methods described in 50.

#### Yeast chronological life span determinations

Most chronological life span determinations were performed in cells grown in liquid synthetic complete media containing 2% glucose (WOGLU) supplemented with standard amounts of amino acids and nucleotide bases as previously described including a four fold excess of the supplements tryptophan, leucine, histidine, methionine, adenine and uracyl to avoid possible artifacts due to the auxotrophic deficiencies of the strains 50. For growth in respiratory media, glucose was substituted by EG (2% ethanol and 2% glycerol). For calorie restriction (CR) experiments, glucose concentration was reduced to 0.5%. Briefly, yeast strains from frozen stock (-80°C) were patched onto complete YPD (2% glucose) or YPEG (2% ethanol and 2% glycerol) agar plates and incubated at 30°C. The following day, cells were inoculated into 10 ml of minimum WO media with the appropriate carbon sources and grown overnight. After 24 hours, cells were inoculated into 50 ml of WO media in 250-ml flasks to an optical density at 600 nm (OD^600^) of 0.250. Cultures were grown with shaking (250 rpm) at 30°C. Maximum cell density is normally reached after 72 hours of growth in WO, therefore 3 days after inoculation was considered as “day 0” of chronological life span. Subsequently, cellular viability was determined at the indicated days by either propidium iodide (PI) staining followed by flow cytometry analysis or by the colony formation unit (CFU) assay. 

#### Propidium iodide staining and flow cytometry analysis

Membrane integrity as a marker of cell viability was assessed by PI staining (Molecular Probes, Eugene, OR, USA) and analyzed by flow cytometry as previously described 50. Every other day, a sample of stationary phase cultures containing 10^6^ yeast cells per ml in phosphate-buffered saline (PBS) was incubated for 30 min at 30 °C in the presence of 2 μM PI. Flow cytometry analysis was performed on a Becton Dickinson (BD) LSRFortessa™ cell analyzer as described 50. Excitation was performed using a yellow/green laser at 561 nm; emission was detected using a 20 nm bandpass filter centered at 660 nm (Becton Dickinson, NJ, USA). For each yeast population, three samples of ten thousand cells were analyzed.

#### Colony formation unit assay

To perform the colony formation unit (CFU) assay, cell number was estimated by optical density (OD) for each population and serial dilutions of different cultures were plated onto 3 or 4 YPD plates at an approximate concentration of 100 cells per plate. Plates were incubated at 30°C for 48 h and CFU were counted.

#### Fluorescence microscopy

Wide-field fluorescence microscopy for verification of polyQ-GFP expression after β-estradiol induction in yeast was performed as described 4. Briefly, we used an Olympus fluorescence BX61 microscope equipped with Nomarski differential interference contrast (DIC) optics, a Uplan Apo 100× objective (NA 1.35), a Roper CoolSnap HQ camera, and Sutter Lambda10-2 excitation and emission filter wheels, and a 175 W Xenon remote source with liquid light guide. Briefly, PI-stained cells were mounted on to slides and examined using a CY3 filter. Images were acquired using SlideBook 4.01 (Intelligent Imaging Innovations, Denver, CO, USA).

#### Cell respiration

Assessment of yeast endogenous cellular respiration was performed polarographically using a Clark-type oxygen electrode (Hansatech Instruments, Norfolk, UK) at 30°C as described 51. Measurement of respiration in the exponential phase of growth was performed 16 h after concomitant strain inoculation and induction. For exponential phase respiration, cells were grown overnight in the indicated media, reinoculated in fresh media at the same confluence (OD^600^ = 0.250) and grown for 3-6 h, as indicated, to measure maximal cellular respiration. For stationary phase respiration, cell were treated as describe above and grown for 72 h (day 0) before measure respiration, to allow cell to reach stationary phase. The specific activities reported were corrected for KCN-insensitive respiration.

#### Chemical treatments

Serial dilution growth tests were performed in solid media containing glucose, raffinose or galactose as the carbon source, supplemented or not with several mitochondrial poisons. Plates were supplemented with either 50 μM antimycin A (Sigma) to inhibit the mitochondrial respiratory chain complex III or 10 μM oligomycin (Sigma) to inhibit the mitochondrial F_1_F_0_ ATP synthase. Plates were also supplemented with increasing concentrations (10-40 μM) of the uncoupler CCCP (carbonyl cyanide m-chlorophenyl hydrazine). In the “untreated” plates, an equal volume of drug vehicle (ethanol) was added as a control. 

### Drosophila strains and methods

#### Drosophila strains and culture conditions

All fly strains were maintained on a cornmeal–molasses–yeast medium at room temperature (~22°C) with 60–65% humidity. The following *Drosophila* strains were used in the studies: *elav^C155-GAL4^* and *UAS-CD8GFP* were obtained from Bloomington *Drosophila* Stock Center. *UAS-hHttExQ20* and *UAS-hHttExQ93* were obtained from Dr. Leslie Thompson 33. *UAS-DmPGC-1α* (*UAS-srl*) was obtained from Dr. Christian Frei 52.

#### Negative geotaxis behavior assay

Ten age-matched female flies from each genotype were placed in a vial marked with a circle 8 cm above the bottom surface. The flies were gently tapped to the bottom and given 10 s to climb. After 10 s, the number of flies that successfully climbed above the 8 cm mark was recorded and divided by the total number of flies. The assay was repeated 10 times and the data were represented as the averaged percentages. Ten independent groups (total 100 flies) per genotype was tested 53.

#### Protein extraction and western blot analysis

Proteins were extracted from fly heads using the homogenizing buffer containing 10 mM HEPES pH 7.9, 1.5 mM MgCl_2_, 10 mM KCl, 1 mM dithiothreitol (DTT) and 0.5 mM phenylmethylsulphonyl fluoride (PMSF). Samples were then spun at 14,000g for 30 min at 4°C and the supernatant was collected as supernatant 1. The pellets were resuspended with the protein extraction buffer containing 30 mM HEPES pH 7.9, 0.6 M NaCl, 1.5 mM MgCl_2_, 0.4 mM EDTA, 1 mM DTT, 25% glycerol, 1% NP-40 and 0.5 mM PMSF and incubated at 4°C for 30 min with vortexing every 6 min. Then the supernatant 2 was collected after 30 min centrifugation at 14,000g at 4°C. Next, supernatants 1 and 2 were equally mixed and heated at 95°C with 4 × Laemmli sample buffer 54. Samples were loaded onto 15% SDS-polyacrylamide gels and transferred to nitrocellulose membranes. Blots were probed with anti-ATP5α antibody (1:10,000, Abcam), anti-cleaved caspase 3 (1:1,000, Cell Signaling), or anti-β-Actin (1:10,000, Sigma) primary antibodies, and then probed with infrared dye-conjugated IR700 and IR800 secondary antibodies (1:10,000, LI-COR Biosciences). Blots were imaged and processed on an Odyssey Infrared Imaging system. 

#### Immunohistochemistry of fly brains

Adult brains were dissected in ice cold PBS (pH 7.4), fixed in 4% formaldehyde for 15 min and washed in PBS with 0.4% Triton X-100 (PBTX). Brains were incubated at 4°C overnight with the following antibodies diluted in PBTX with 5% normal goat serum: anti-cleaved caspase 3 antibody (Asp175; 1:250, Cell Signaling); secondary antibody conjugated to Alexa 488 (1:250, Jackson ImmunoResearch, Molecular Probes). Tissues were then mounted on microscope slides in Vectashield Mounting Medium for Fluorescence (Vector Laboratories).

#### Confocal image acquisition and processing

Confocal microscopy was performed with an Olympus IX81 confocal microscope using an Olympus PlanApo N 20x objective. Images from different genotypes were captured using the same scan setting and parameters. Images were processed using FluoView 10-ASW (Olympus) and assembled using Adobe Photoshop CS6 (Adobe Systems).

### Statistical Analysis

All experimental measurements were done at least in triplicate. All results are presented as means ± S.E.M. or S.D. as appropriate. Significance testing was performed using the Student’s T-test or One-Way ANOVA, as noted in the figure legends.

## SUPPLEMENTAL MATERIAL

Click here for supplemental data file.

All supplemental data for this article are also available online at http://microbialcell.com/researcharticles/attenuation-of-polyglutamine-induced-toxicity-by-enhancement-of-mitochondrial-oxphos-in-yeast-and-fly-models-of-aging/.
