# Supplementary material for: Attenuation of polyglutamine-induced toxicity by enhancement of mitochondrial OXPHOS in yeast and fly models of aging
Source: Microb Cell. 2016 Jul 26;3(8):338–51. doi: 10.15698/mic2016.08.518 (PMC5349013; doi:10.15698/mic2016.08.518)

SUPPLEMENTARY MATERIAL

Supplemental Figure 1. Suppression of polyQ-induced toxicity by *HAP4*-overexpression induced enhancement of mitochondrial biogenesis estimated by the colony formation units (CFU) assay. Yeast CLS. Survival was estimated from the number of colonies formed from ~150 cells of each strain plated on non-inducing media (YPD) after 2 days of growth at 30°C. Error bars are S.D, n=3.

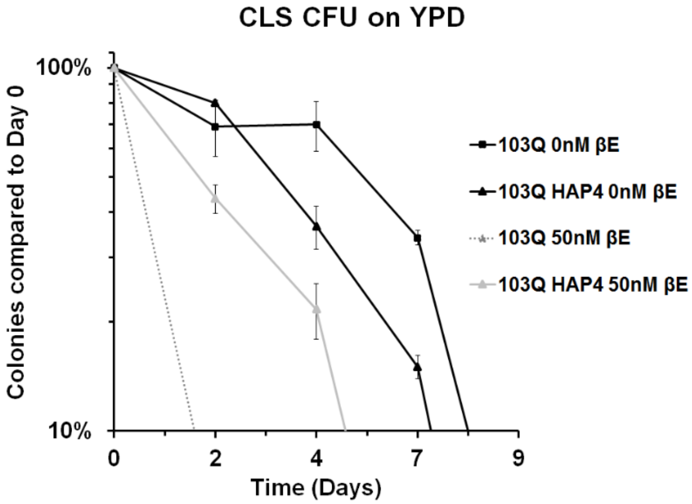

Supplement: Supplementary file 1 [file mic-03-338-s01.pdf]
